# Impact of sarcopenia on outcomes of patients undergoing liver resection for hepatocellular carcinoma

**DOI:** 10.1002/jcsm.13040

**Published:** 2022-07-19

**Authors:** Jinhuan Yang, Kaiwen Chen, Chongming Zheng, Kaiyu Chen, Jian Lin, Qishan Meng, Ziyan Chen, Liming Deng, Haitao Yu, Tuo Deng, Zhiyuan Bo, Qikuan He, Yi Wang, Gang Chen

**Affiliations:** ^1^ Department of Hepatobiliary Surgery The First Affiliated Hospital of Wenzhou Medical University Wenzhou Zhejiang China; ^2^ The First School of Medicine, School of Information and Engineering Wenzhou Medical University Wenzhou Zhejiang China; ^3^ School of Ophthalmology and Optometry, School of Biomedical Engineering Wenzhou Medical University Wenzhou Zhejiang China; ^4^ Department of Epidemiology and Biostatistics, School of Public Health and Management Wenzhou Medical University Wenzhou Zhejiang China; ^5^ Key Laboratory of Diagnosis and Treatment of Severe Hepato‐Pancreatic Diseases of Zhejiang Province The First Affiliated Hospital of Wenzhou Medical University Wenzhou Zhejiang China

**Keywords:** Sarcopenia, Hepatocellular carcinoma, Muscle strength, Muscle mass, Liver resection

## Abstract

**Background:**

Previous studies have indicated that sarcopenia is associated with poor post‐operative outcomes in liver cancer patients, but the studies are limited by confounding from mixed diseases, retrospective data, and non‐standardized measurement methods. At present, there is no research with both muscle mass and strength as predictors for hepatocellular carcinoma (HCC) outcomes. We studied the impact of sarcopenia on post‐operative outcomes in HCC patients in a cohort study designed according to the European Working Group on Sarcopenia in Older People standards.

**Methods:**

A total of 781 consecutive patients admitted to our centre were registered from May 2020 to August 2021. All participants submitted questionnaires and underwent handgrip strength, chair stand test, physical performance, and computed tomographic evaluation. Then, they were divided into three groups according to muscle mass and strength: Group A (reduced muscle mass and strength), Group B (reduced muscle strength or reduced muscle mass), and Group C (normal muscle mass and strength). The baseline data and post‐operative outcomes were compared and analysed. The primary outcome variable in this study was the presence of a major post‐operative complication, and the secondary outcome was the 90‐day re‐admission rate.

**Results:**

A total of 155 patients [median age, 60.00 (IQR, 51.00–66.00) years; 20 females (12.90%)] were included after strict exclusion. The mean (SD) BMI was 23.37 ± 0.23 kg/m^2^. The mean (SD) SMI of all participants was 47.05 ± 0.79 cm^2^/m^2^, and the mean (SD) handgrip strength was 32.84 ± 0.69 kg. Among them, 77 (49.68%) patients underwent laparoscopic hepatectomy, and 73 (47.10%) patients received major hepatectomy. Regarding the post‐operative results, Group A had a higher rate of major complications [40.91% (9 of 22) vs. 11.94% (8 of 67) in Group B and 6.06 (4 of 66) in Group C; *P* = 0.001], higher rate of blood transfusion (77.27% vs. 46.27% in Group B and 42.42% in Group C; *P* = 0.015), higher hospitalization expenses (*P* = 0.001), and longer hospital stay (*P* < 0.001). There was no difference in 90‐day re‐admission rates among the three groups. Sarcopenia (hazard ratio, 10.735; 95% CI, 2.547–45.244; *P* = 0.001) and open surgery (hazard ratio, 4.528; 95% CI, 1.425–14.387; *P* = 0.010) were independent risk factors associated with major complications.

**Conclusions:**

Sarcopenia is associated with adverse outcomes after liver resection for HCC. It should be evaluated upon admission to classify high‐risk patients and reduce the risk of major complications.

## Introduction

Liver cancer is the sixth most commonly diagnosed cancer worldwide and the third leading cause of cancer‐related death,^1^ with the most common primary hepatic malignancy being hepatocellular carcinoma (HCC). Currently, surgical resection is still the primary treatment for liver cancer,^2^, ^3^ and many factors have been associated with the short‐term and long‐term post‐operative outcomes in these patients. An increased number of hepatic segments resected,^4^, ^5^ increased operative blood loss,^4^, ^6^, ^7^ severe liver cirrhosis,^8^, ^9^ and muscle depletion (reduced muscle mass or strength)^10^, ^11^, ^12^ lead to reduced survival; blood transfusion^9^ and a longer operative time^13^ increase the risk of post‐operative complications; and general anaesthesia, high doses of opiates, and open surgery increase the possibility of post‐operative recurrence.^14^, ^15^ Some factors affecting post‐operative outcomes, such as the number of hepatic segments resected, operative blood loss, and the severity of liver cirrhosis, are objective factors for which post‐operative interventions are difficult to administer. Therefore, it is more practical to identify strong prognostic predictors during the pre‐operative evaluation, which can help to improve clinical management during the perioperative period.

Sarcopenia is a progressive systemic skeletal muscle disease that includes a decrease in muscle strength and a decrease in muscle mass.^16^ Studies have found that sarcopenia is associated with poorer outcomes in patients with chronic respiratory disease,^17^ liver cirrhosis,^18^ lung cancer,^19^ liver cancer,^20^ and colorectal cancer.^21^ Patients with sarcopenia have a higher risk of falls and fractures,^16^ a higher cancer recurrence rate,^20^ lower overall survival,^18^ and more severe complications^19^ after surgery. Patients who undergo major abdominal surgery experience further loss in muscle mass, which can lead to worse post‐operative outcomes.^22^, ^23^ Some studies have shown that pre‐operative exercise,^24^ nutritional intervention,^25^ supplementation with leucine,^26^ etc., can decrease sarcopenia, improving short‐term post‐operative outcomes in patients with sarcopenia. Therefore, if we can accurately detect or identify patients with sarcopenia at the first clinical encounter, it is expected that intervention measures could be applied before surgery to improve post‐operative outcomes.

Although previous studies could distinguish sarcopenic and non‐sarcopenic liver cancer patients using imaging indicators and scholars also noted that this classification approach has obvious discrimination, there are disadvantages in the use of retrospective data, such as small sample size, mixed disease, and lack of assessment of muscle strength,^12^, ^27^, ^28^ resulting in poor clinical applicability for specific diseases. The recent EWGSOP (European Working Group on Sarcopenia in Older People) expert consensus states that muscle strength is better for predicting adverse outcomes than muscle mass.^16^ Moreover, no prospective databases have focused on HCC patients (*Table* S1). In other words, based on the latest EWGSOP definition,^16^ there is no research on how to accurately target specific types of liver cancer by combining muscle mass and strength. Therefore, this cohort study aimed to investigate the effect of sarcopenia as an independent factor in patients with primary HCC after liver resection under the latest EWGSOP definition.^16^

## Methods

A total of 781 patients who received consecutive treatment at our study centre were prospectively registered from May 2020 to August 2021. All patients accepted the SARC‐F questionnaire, lifestyle questionnaire, muscle strength test (grip strength and chair stand test), physical performance (gait speed), and imaging evaluation following the EWGSOP standard when first admitted to the hospital did not receive any treatment.^16^ Patient demographics, co‐morbidity, ASA score, first haematological index after admission (before taking medication), type of surgical approach and type of resection performed, intra‐operative data (blood loss, blood transfusions, and operative time), tumour‐related factors [type of tumour, tumour size, number of tumours, microvascular invasion (MVI), degree of differentiation, TNM stage, presence of satellite nodules, lymph node metastases, and tumour markers], post‐operative outcomes (haematological index on the first and seventh day after operation, blood transfusions, complication, hospital stay, hospitalization expenses, and 90‐day re‐admission rate), etc., were recorded. The prospective database was then used for retrospective analysis.

**Figure 1:**
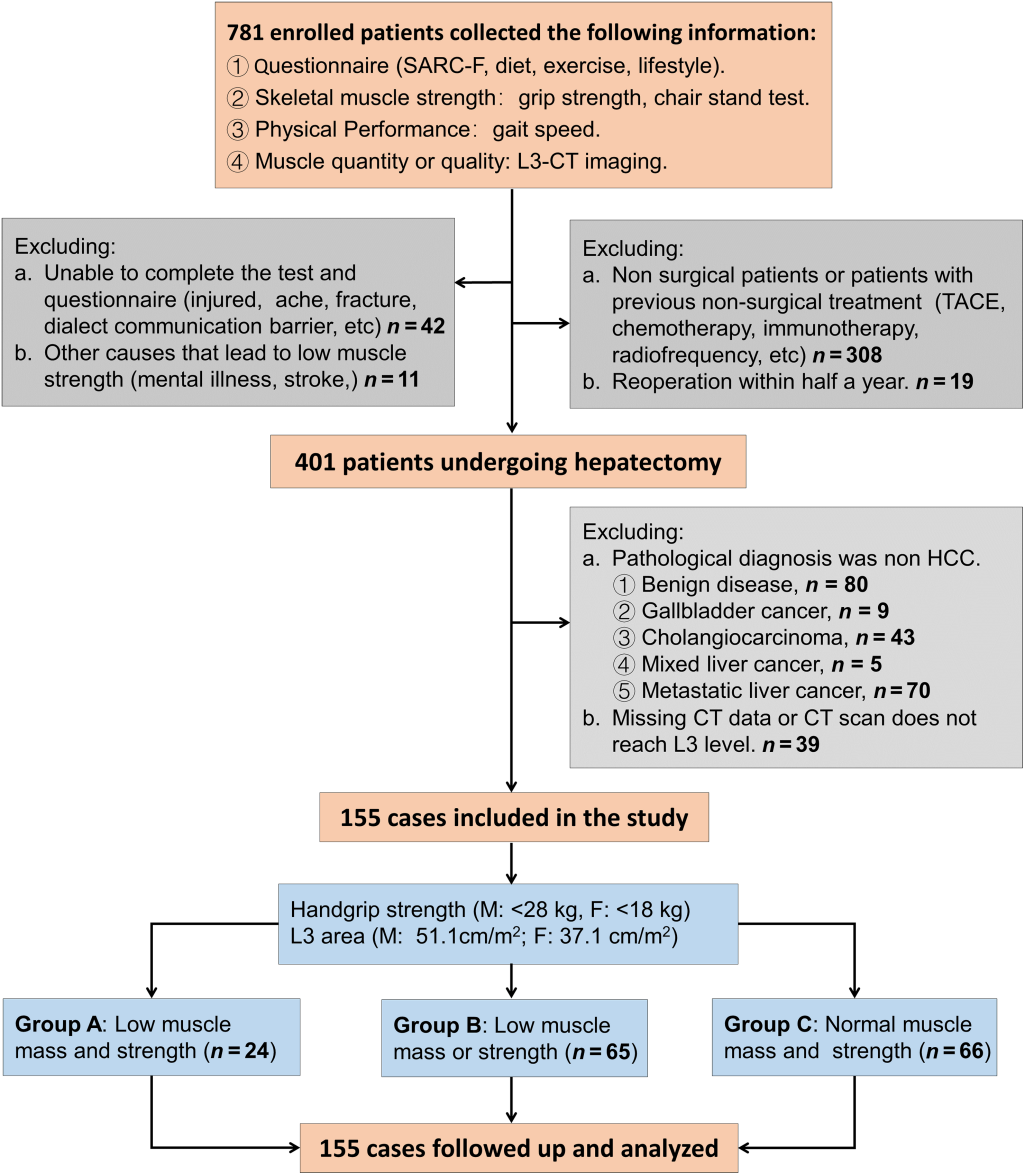
Flow chart of research design.

The patients were further screened according to the following exclusion criteria: (i) those who did not accept liver resection, (ii) those who were diagnosed with non‐HCC (benign lesions, metastatic liver cancer, and mixed liver cancer) by pathology, (iii) those who had other concomitant pre‐operative causes of muscle weakness (injury, fracture, stroke, etc.), (iv) those who missing CT data or CT scans did not reach the level of the third lumbar vertebra (L3), (v) those who accepted adjuvant therapy such as chemotherapy and abdominal surgery within 1 year before surgery, and (vi) those who could not complete the standard tests and questionnaires (*Figure* 1).

The primary outcome of this cohort study was post‐operative complications. The 90‐day re‐admission rate was investigated as a secondary outcome. We obtained written informed consent from each patient before the research.

The study protocol (*Data* S1) was approved by Ethics Committee in Clinical Research of the First Affiliated Hospital of Wenzhou Medical University.

### Definitions

HCC was graded according to the 8th Edition American Joint Committee on Cancer staging definition for hepato‐pancreato‐biliary cancer.^[S1]^. Patient co‐morbidities were graded using the age‐adjusted Charlson comorbidity score.^[S2]^ Liver function and short‐term survival rates were evaluated with the MELD classification, BCLC, and Child–Pugh score.^[S3, S4]^ SARC‐F scale scores ranged from 0 to 10 (i.e. 0–2 points for each component; 0 = *best* to 10 = *worst*) and were dichotomized to represent symptomatic (≥4) vs. healthy (<4) status.^[S5]^ Physical activity was defined as moderate‐to‐high intensity exercise performed for ≥1–2 h/week or light‐intensity exercise performed for >4 h/week.^[S6]^ Patients were divided into groups based on their diet: vegetarian (excluding meat, poultry, and fish in the diet), semi‐vegetarian (excluding red meat in the diet), and meat eater (including red meat in the diet).^[S7]^ Complications were defined according to the Clavien–Dindo classification.^[S8]^ Grade 1–2 complications were defined as minor complications and included wound infection (bedside), nausea, vomiting, and elevated blood pressure; Grade 3 or higher complications were defined as major complications and included post‐operative pleural effusion (excluding reactive pleural effusion in patients undergoing right liver resection), bile leakage, post‐operative bleeding, liver failure, and death. Bile leakage, post‐operative bleeding, and liver failure post‐hepatectomy were assessed according to the International Study Group of Liver Surgery.^[S9–11]^ Removing three or more segments and laparoscopic resections of posterior superior segments were considered major resections.^[S12]^

### Characteristic parameters of sarcopenia

**Figure 2:**
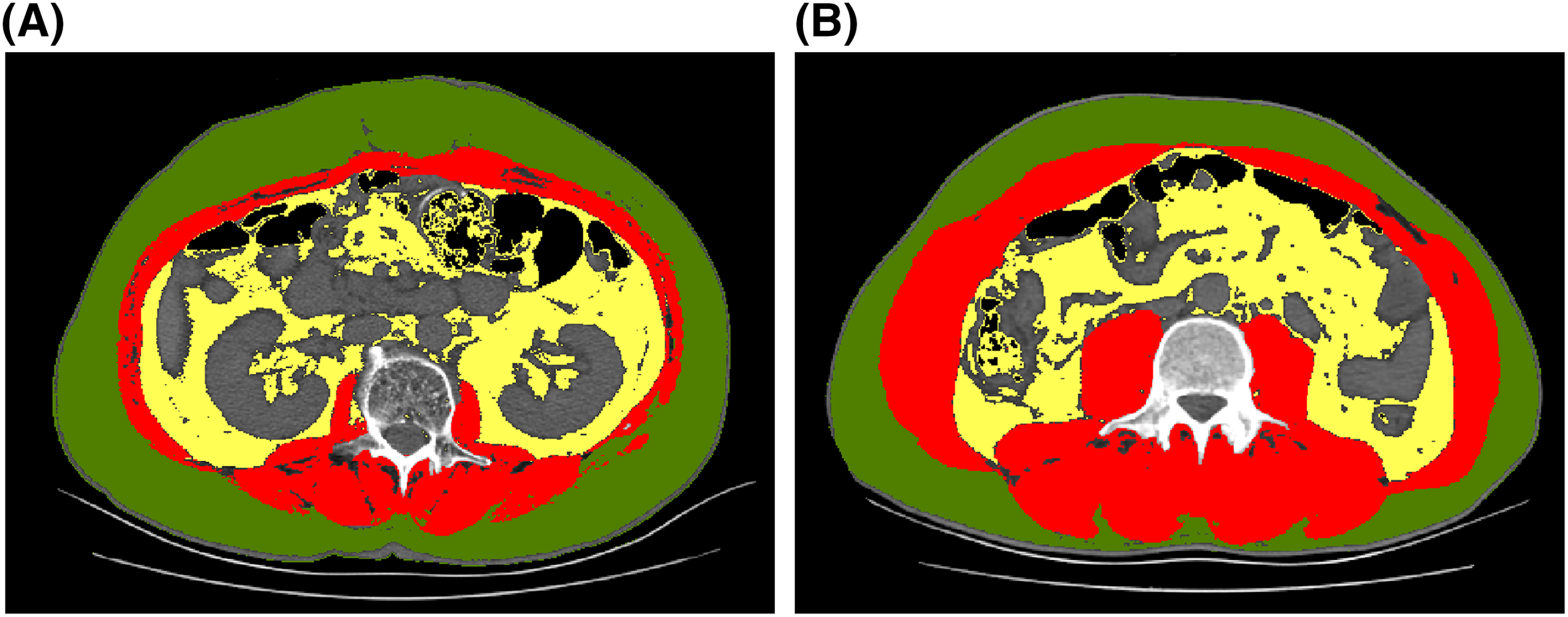
Computed tomographic scans showing areas of skeletal muscle (red), visceral adipose tissue (yellow), and subcutaneous adipose tissue (green) in patients with (A) and without sarcopenia (B).

Two researchers independently measured the total cross‐sectional skeletal muscle area, visceral adipose tissue area, and subcutaneous fat area at level L3 for each patient in a blinded fashion on the most recent pre‐operative CT image (image and surgery time interval within 7 days) to obtain the mean skeletal muscle cross‐sectional area, mean visceral fat area, and mean subcutaneous fat area. Skeletal muscle, subcutaneous adipose tissue, and visceral adipose tissue were identified and quantified in Hounsfield units (HU) using ImageJ 1.53e (*Figure* 2). A threshold range of −29 to 150 HU was used to define skeletal muscle, a range of −150 to −50 HU was used to define visceral adipose tissue, and a range of −190 to −30 HU was used to define subcutaneous adipose tissue. BMI was calculated as weight (kg)/height (m^2^). SMI was calculated as the total cross‐sectional skeletal muscle area in the L3 plane (cm^2^) /height (m^2^). SMI is an internationally recognized gold standard for assessing sarcopenia,^16^ and a low SMI is considered a sign of low muscle mass. Therefore, in this research, we used SMI as an indicator to judge patients' muscle mass. When the patient was admitted but did not receive any invasive treatment, muscle strength, including the grip strength test,^16^, ^29^ chair stand test,^16^ and physical performance assessment (gait speed test^16^), was assessed. In the grip strength test, the dominant hand and the nondominant hand were measured twice intermittently (kg), and an average value of four values was obtained. The latest data from the Asian sarcopenia grip test indicated that 28 kg and 18 kg were the critical values for males and females, respectively.^30^ Based on the ROC curve of SPSS, cut‐off values for SMI were defined as 51.1 cm^2^/m^2^ in males and 37.2 cm^2^/m^2^ in females. The chair stand test was administered, and the time required for the patient to stand five times from a sitting position without using the arms was measured. For the gait speed test, the time patients spent walking 8 m on a flat indoor floor at their usual walking speed was measured.

Finally, we divided the patients into three groups according to their muscle strength (grip strength) and muscle mass (SMI): Group A (low muscle strength and low muscle mass), Group B (low muscle strength or low muscle mass), and Group C (normal muscle strength and normal muscle mass).

### Statistical analysis

The Kolmogorov–Smirnov test and the Shapiro–Wilk test were used to assessing the distribution of variables. We used the mean (SD) and the median [interquartile range (IQR)] to express parametric continuous variables and non‐parametric distribution of data, respectively.

If the variables conformed to a normal distribution, an analysis of variance was used for comparisons. If not, the Kruskal–Wallis test was used. When the variable was categorical, we used the χ2 test or Fisher's exact test with Yates correction to compare differences. If there were statistically significant differences among the three groups, we used Bonferroni correction to perform a post hoc analysis.

**Figure 3:**
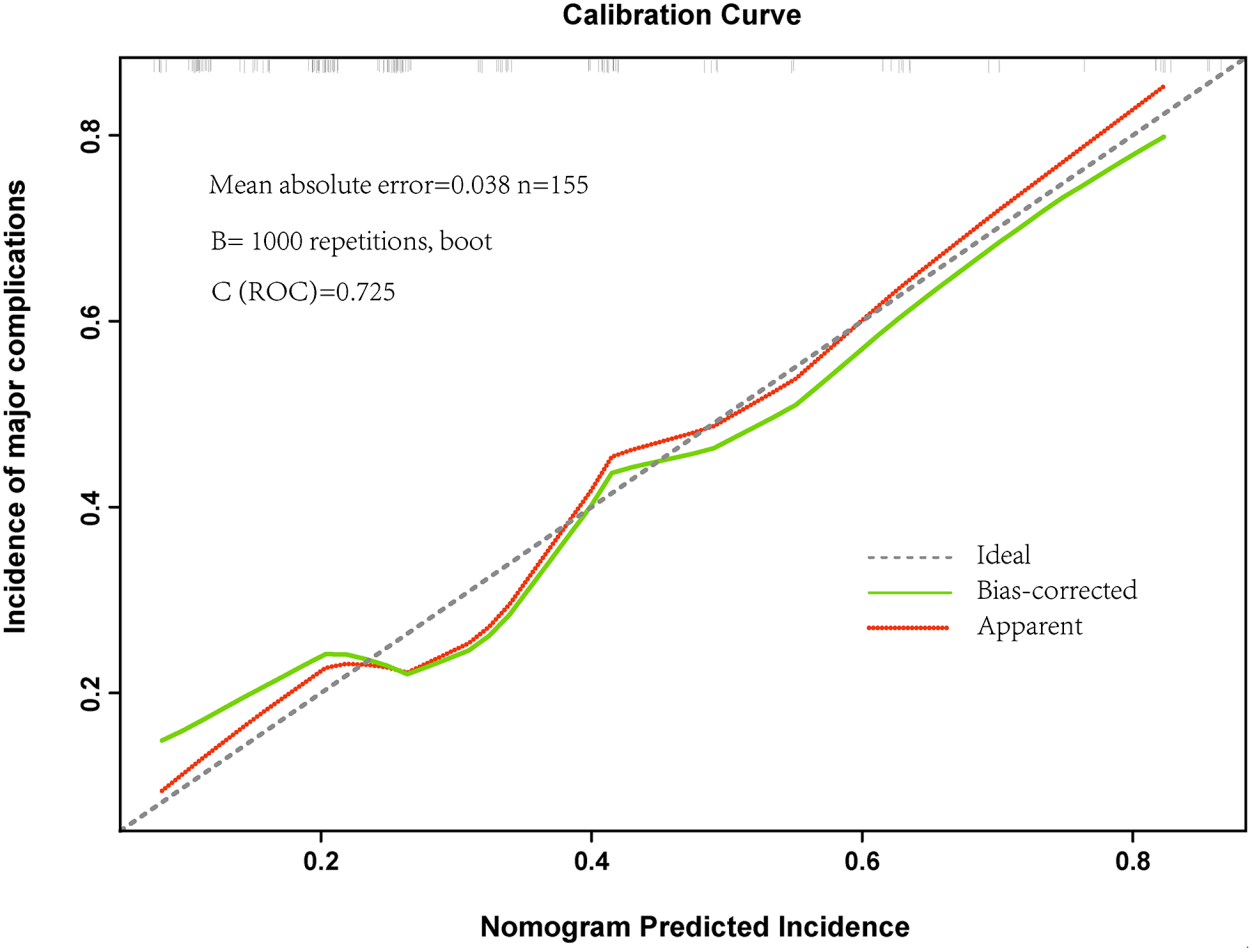
Calibration plots for pre‐operative factors models associated with the prediction of the complication incidence.

We performed multivariate logistic regression on risk factors that were statistically significant in the univariate analysis and several universally acknowledged factors to predict the occurrence probability of major complications (*Figure* 3). We used SPSS software, Version 21.0 (IBM Corp) for MacOSX, to perform statistical analysis and R (4.1.2) to draw the calibration curve in this study. Two‐sided *P* < .05 was considered statistically significant.

## Results

A total of 155 patients [median age, 60.00 (IQR, 51.00–66.00) years; 20 females (12.90%)] were included after strict exclusion (*Figure* 1). The baseline characteristics of the included population are provided in *Table* 1. The mean (SD) BMI was 23.37 (0.23) kg/m^2^, and the median abdominal circumference was 87.20 (IQR, 83.00–91.00) cm. Among them, two (1.29%) had a SARCF score ≥4, and 100 patients (64.52%) were diagnosed with liver cirrhosis. In this cohort, 148 patients (95.48%) had Child–Pugh Stage A disease, and 138 patients (89.03%) had TNM Stage I–II disease.

**Table 1:** Baseline characteristics of patients based on muscle mass and muscle strength

	Total (*n* = 155)	Group A (*n* = 22)	Group B (*n* = 67)	Group C (*n* = 66)	*P* value
Age, y	60.00 (51.00–66.00)	63.00 (54.05–70.00)	63.00 (54.00–67.00)	58.00 (49.75–65.00)	0.106
Sex, *n*					0.059
Female	20 (12.90)	4 (18.18)	4 (5.97)	12 (18.18)	
Male	135 (87.10)	18 (81.82)	63 (94.03)	54 (81.82)	
BMI, kg/m^2^	23.37 ± 0.23	21.80 ± 0.46	22.40 ± 0.35	24.80 ± 0.30	<0.001
Abdominal circumference, cm	87.20 (83.00–91.00)	86.00 (80.50–87.40)	87.20 (82.00–90.00)	87.20 (85.00–95.25)	0.017
SARCF, *n* (%)					<0.001
>=4	2 (1.29)	2 (9.09)	0 (0)	0 (0)	
<4	153 (98.71)	20 (90.91)	67 (100)	66 (100)	
Grip strength, kg	32.84 ± 0.69	21.60 ± 1.09	34.40 ± 0.75	35.00 ± 1.08	<0.001
Chair stand test, s	13.62 (11.61–15.87)	16.42 (13.83–19.15)	13.40 (11.35–14.95)	13.65 (11.30–15.14)	0.001
Gait speed, m/s	1.07 (0.96–1.16)	1.02 (0.84–1.14)	1.07 (0.90–1.19)	1.07 (0.98–1.15)	0.653
SMI, cm/m^2^	47.05 ± 0.79	42.20 ± 1.55	41.80 ± 0.93	54.00 ± 1.04	<0.001
Visceral adipose tissue, cm^2^	112.18 (98.72–144.01)	102.68 (59.07–138.29)	103.54 (61.23–127.14)	144.01 (116.69–164.63)	<0.001
Subcutaneous adipose tissue, cm^2^	113.23 (94.94–140.75)	113.72 (92.06–151.06)	103.37 (82.58–113.23)	137.91 (112.48–145.83)	<0.001
ASA grade, *n* (%)					<0.001
I	11 (7.10)	2 (1.29)	6 (8.95)	3 (4.54)	
II	125 (80.65)	16 (72.73)	47 (70.15)	62 (93.94)	
III	19 (12.26)	4 (18.18)	14 (20.90)	1 (1.52)	
Cirrhosis, *n* (%)	100 (64.52)	16 (72.73)	42 (62.69)	42 (63.64)	0.681
Ascites, *n* (%)	12 (7.74)	5 (22.73)	4 (5.97)	3 (4.55)	0.036
Hypertension, *n* (%)	50 (32.26)	6 (27.27)	23 (34.33)	21 (31.82)	0.824
Diabetes, *n* (%)	34 (21.94)	8 (36.36)	11 (16.42)	15 (22.73)	0.140
Age‐adjusted Charlson co‐morbidity index score	6.00 (5.00–7.00)	6.00 (5.00–7.00)	6.00 (6.00–7.00)	6.00 (5.00–7.00)	0.244
Child–Pugh stage, *n* (%)					0.121
A	148 (95.48)	19 (86.36)	65 (97.01)	64 (96.97)	
B	7 (4.52)	3 (13.64)	2 (2.99)	2 (3.03)	
MELD score	5.00 (4.00–7.00)	5.50 (4.00–6.25)	5, 00 (3.00–7.00)	5.00 (4.00–6.00)	0.325
BCLC stage, *n* (%)					0.185
0	20 (12.90)	2 (9.09)	13 (19.40)	5 (7.58)	
A	85 (54.84)	13 (59.12)	31 (46.31)	41 (62.10)	
B	5 (3.23)	2 (9.09)	2 (2.99)	1 (1.52)	
C	45 (29.03)	5 (22.70)	21 (31.30)	19 (28.80)	
TNM stage					0.468
I–II	138 (89.03)	18 (81.82)	61 (91.04)	59 (89.39)	
III–IV	17 (10.97)	4 (18.18)	6 (8.96)	7 (10.61)	
Microvascular invasion, *n* (%)	16 (10.32)	2 (9.09)	9 (13.40)	6 (9.09)	0.780
Satellite stove, *n* (%)	15 (9.68)	1 (4.55)	9 (13.40)	5 (7.58)	0.483
Lesion size, cm	3.20 (2.20–5.00)	3.95 (2.88–5.60)	3.41 (2.00–4.00)	3.40 (2.50–5.18)	0.023
Lesions, *n* (%)					0.780
Solitary	138 (89.03)	20 (90.91)	58 (86.57)	60 (90.91)	
Multiple	17 (10.97)	2 (9.09)	9 (13.43)	6 (9.09)	
Differentiation of HCC, *n* (%)					0.385
Poor	42 (27.10)	6 (27.27)	17 (25.38)	19 (28.78)	
Moderate	93 (60.00)	12 (54.55)	45 (67.16)	36 (54.55)	
Well	20 (12.90)	4 (18.18)	5 (7.46)	11 (16.67)	
Smoking, *n* (%)	98 (63.23)	13 (59.09)	44 (65.67)	41 (62.12)	0.832
Drinking, *n* (%)	87 (56.13)	12 (54.55)	39 (58.21)	36 (54.55)	0.902
Physical activity, *n* (%)	101 (65.16)	11 (50.00)	47 (70.15)	43 (65.15)	0.227
Dietary structure, *n* (%)					0.014
Semi‐vegetarian	59 (38.06)	6 (27.27)	30 (44.78)	23 (34.85)	
Meat eater	63 (40.65)	5 (22.73)	28 (41.79)	30 (45.45)	
Vegetarian	33 (21.29)	11 (50.00)	9 (13.43)	13 (19.70)	
Sleep time, *n* (%)					0.248
>=8 h	35 (22.58)	8 (36.36)	13 (19.40)	14 (21.21)	
<8 h	120 (77.42)	14 (63.64)	54 (80.60)	52 (78.79)	

ASA, American Society of Anesthesiologists; BMI, body mass index; MELD, model for end‐stage liver disease.

The included patients were then divided into three groups according to the SMI and grip strength. For muscle mass, Group A (low muscle mass and low muscle strength) had a statistically significantly lower SMI [mean (SD), 42.20 (1.55) vs. 41.80 (0.93) cm^2^/m^2^ in Group B and 54.00 (1.04) cm^2^/m^2^ in Group C; *P* < 0.001] than Group B and Group C. Meanwhile, Group A had a lower abdominal circumference [86.00 (IQR, 80.50–87.40) vs. 87.20 (IQR, 82.00–90.00) cm in Group B and 87.20 (IQR, 85.00–95.25) cm in Group C; *P* = 0.017] and lower BMI [mean (SD), 21.80 (0.46) vs. 22.40 (0.35) kg/m^2^ in Group B and 24.80 (0.30) kg/m^2^ in Group C; *P* < 0.001]. In terms of muscle strength, Group A had weaker handgrip strength [mean (SD), 21.60 (1.09) vs. 34.40 (0.75) kg in Group B and 35.00 (1.08) kg in Group C; *P* < 0.001] and a worse chair stand test [16.42 (IQR, 13.83–19.15) vs. 13.40 (IQR, 11.35–14.95) s in Group B and 13.65 (IQR, 11.30–15.14) s in Group C; *P* = 0.001] than Group B and Group C. In addition, the visceral adipose tissue [102.68 (IQR, 59.07–138.29) in Group A vs. 103.54 (IQR, 61.23–127.14) cm^2^ in Group B and 144.01 (IQR, 116.69–164.63) cm^2^ in Group C; *P* < 0.001] and subcutaneous adipose tissue [113.72 (IQR, 92.06–151.06) in Group A vs. 103.37 (IQR, 82.58–113.23) cm^2^ in Group B, and 137.91 (IQR, 112.48–145.83) cm^2^ in Group C; *P* < 0.001] were significantly different among the groups. According to the questionnaire results, Group A had a higher proportion of vegetarians [50.00% (11 of 22) in Group A vs. 13.43% (9 of 67) in Group B and 19.70% (13 of 66) in Group C; *P* = 0.014] than Group B and Group C. Most haematological indicators of the first admission (*Table* S2) were similar among the three groups, except for albumin level (*P* = 0.025), neutrophils (*P* = 0.046), and haemoglobin level (*P* = 0.041). No differences were observed in terms of gait speed, cirrhosis, age‐adjusted Charlson co‐morbidity index score, or MELD score.

In total, 73 patients (47.10%) underwent major hepatectomy, and 78 patients (50.32%) underwent open surgery. The rate of blood loss (≥400 mL) was higher in Group A than in the other groups [36.36% (8 of 22) in Group A vs. 10.45% (7 of 67) in Group B and 9.09% (6 of 66) in Group C; *P* = 0.003]. Regarding the post‐operative results (Table 2), 21 patients had major complications (13.55%), and 76 patients (49.03%) received blood transfusions after surgery. Within 90 days after discharge, 14 patients (9.03%) were hospitalized again. Specifically, Group A had a statistically significant difference in major complications [40.91% (9 of 22) in Group A vs. 11.94% (8 of 67) in Group B and 6.06% (4 of 66) in Group C; *P* < 0.001], a higher transfusion ratio [77.27% (17 of 22) in Group A vs. 46.27% (31 of 67) in Group B and 42.42% (28 of 66) in Group C; *P* = 0.015], longer hospital stay [14.50 (IQR, 10.75–20.50) in Group A vs. 12.00 (IQR, 9.00–14.00) d in Group B and 10.50 (IQR, 9.00–13.00) d in Group C; *P* = 0.001], and higher hospitalization expenses [57374.12 (IQR, 45104.47–90485.91) in Group A vs. 50987.00 (IQR, 39663.30–63580.67) yuan in Group B and 44874.83 (IQR, 38980.34–57529.52) yuan in Group C; *P* = 0.001] compared with Group B and Group C. Accordingly, the haematological indexes at different time after operation showed that RBC (Days 1 and 7, *P* = 0.003), platelet (Day 7, *P* = 0.037), total bilirubin (Day 7, *P* = 0.015), and ALB (Day 1, *P* < 0.001) level in sarcopenia group were also different from other groups (*Table* S3), which were related to blood loss. No differences were observed in the 90‐day re‐admission rate (*P* = 0.999), type of hepatectomy (*P* = 0.330), operative time (*P* = 0.077), or operation mode (*P* = 0.785), among the groups.

**Table 2:** Perioperative results of patients according to muscle mass and muscle strength

	Total (*n* = 155)	Group A (*n* = 22)	Group B (*n* = 67)	Group C (*n* = 66)	*P* value
Operation mode, *n* (%)					0.785
Laparoscopic	77 (49.68)	11 (50.00)	35 (52.24)	31 (46.97)	
Open	78 (50.32)	11 (50.00)	32 (47.76)	35 (53.03)	
Type of hepatectomy, *n* (%)					0.330
Major	73 (47.10)	13 (59.09)	31 (46.27)	27 (40.91)	
Minor	82 (52.90)	9 (40.91)	36 (53.73)	39 (59.09)	
Operative time, min	176.80 ± 58.90	191.40 ± 67.20	175.50 ± 66.30	166.80 ± 65.00	0.077
Blood loss, median (mL)					0.003
<=400	134 (86.45)	14 (63.64)	60 (89.55)	60 (90.91)	
>400	21 (13.55)	8 (36.36)	7 (10.45)	6 (9.09)	
Major complications (Clavien–Dindo classification III–IV), *n* (%)					0.001
Yes	21 (13.55)	9 (40.91)	8 (11.94)	4 (6.06)	
No	134 (86.45)	13 (59.09)	59 (88.06)	62 (93.94)	
Blood transfusion, *n* (%)	76 (49.03)	17 (77.27)	31 (46.27)	28 (42.42)	0.015
Hospital stay, d	11.00 (9.00–14.00)	14.50 (10.75–20.50)	12.00 (9.00–14.00)	10.50 (9.00–13.00)	<0.001
90‐day re‐admission rate, *n* (%)	14 (9.03)	2 (9.09)	6(8.96)	6 (9.09)	0.999
Hospitalization expenses	48974.00 (40003.00–61182.00)	57374.12 (45104.47–90485.91)	50987.00 (39663.30–63580.67)	44874.83 (38980.34–57529.52)	0.001

According to the univariable logistic regression results, the following factors were statistically significantly associated with an increased risk of major complications: sarcopenia, open surgery, WBC, and PLT level. In the multivariable analysis, sarcopenia (hazard ratio, 10.735; 95% CI, 2.547–45.244; *P* = 0.001) and open surgery (hazard ratio, 4.528; 95% CI, 1.425–14.387; *P* = 0.010) were independent risk factors associated with major complications (*Table* S4). Calibration plots showed good agreement between the observed outcomes and predicted incidence (*Figure* 3).

## Discussion

To the best of our knowledge, this is the first cohort study based on our prospective kept database to investigate the relationship between sarcopenia and short‐term post‐operative outcomes of patients with HCC under the EWGSOP definition.^16^ Unlike all previous retrospective studies in which sarcopenia was defined by CT imaging, in this study, sarcopenia was defined by standard metrics, namely, considering both muscle mass and strength. The only prospective study in the literature is limited due to the inclusion of patients with different diseases,^20^ including HCC, metastatic liver cancer, cholangiocarcinoma, and gallbladder cancer. Differences in the pathological features and invasiveness of these diseases mean that surgical resection ranges, anatomical resections, and even surgical options (open or laparoscopic) also differ, which results in differences in intraoperative data (operation time, blood loss, etc.) and post‐operative outcomes (complications, blood transfusion, etc.). Generally, cholangiocarcinoma patients are more likely to undergo major liver resection or biliary reconstruction than HCC patients, resulting in more severe post‐operative complications, higher recurrence rates, and lower survival rates. Therefore, the precise location of the disease is beneficial for obtaining disease‐specific and reliable data.

In recent years, studies on sarcopenia have revealed that low muscle strength is a better predictor of poor post‐operative outcomes than low muscle mass.^31^, ^32^ Physical performance has been less predictive value than muscle mass and strength.^20^ The Asian Working Group for Sarcopenia proposed that a Short Physical Performance Battery (SPPB) < 9 points and five sit‐ups (chair stand test) > 12 s can be used instead of pace as a threshold for reflecting the decline in physical performance.^30^ This study found that the grip strength and chair stand test accurately predicted adverse short‐term post‐operative outcomes. However, there was no significant difference in physical performance (gait speed) among the three groups, so it has limited predictive value. At the same time, we found that Group A, which is defined by muscle mass and muscle strength, had a higher incidence of major complications, a higher rate of post‐operative blood transfusion, and longer hospital stays than Group B, which was defined by a single measurement. Therefore, pre‐operative localization by measuring muscle mass and muscle strength can better identify sarcopenic patients in the clinic. The most reliable method is screening and detecting sarcopenia in patients through the F‐A‐C‐S (Find‐cases‐Assess‐Confirm‐Severity) approach is recognized as the most reliable method.^16^ In this study, strictly following the F‐A‐C‐S approach, consecutively admitted patients were first administered the SARC‐F questionnaire and lifestyle questionnaire, followed by the assessment of muscle strength (grip strength test and chair stand test), muscle mass (L3 plane total skeletal muscle area), and physical performance (gait speed test) to detect patients with sarcopenia accurately. Then, we excluded patients who did not undergo surgery and samples that could have impaired muscle mass or strength for other reasons to ensure greater homogeneity and good representativeness of the included samples. Chemotherapy, a history of moderate or major surgery, injury, stroke, etc., can lead to low muscle strength or low muscle mass. Studies have shown that chemotherapy can cause a persistent decrease in muscle mass and strength in cancer patients who lasts for over 4 months and stabilizes after 10 months.^33^, ^34^ Excluding patients with a history of chemotherapy within 1 year from the study cohort can effectively prevent the interference caused by chemotherapy. Similarly, surgery can also cause the loss of muscle mass and muscle strength, such as acute loss of skeletal muscle mass after major hepatectomy^22^; the surgery‐related muscle loss is as high as 7.1% (±5.7%) after liver resection for colorectal liver metastases.^23^ However, there is no relevant evidence regarding whether it can be recovered or how long it would take. Therefore, there is no suitable exclusion criterion for this issue. However, patients with a previous surgical history were excluded from the previous screening. Finally, we screened patients diagnosed with HCC confirmed by pathology among the patients undergoing hepatectomy.

Epidemiological data suggest that the prevalence of sarcopenia increases with age and is more prevalent in men than in women.^[S13, S14]^ Hypertension and diabetes have also been linked to sarcopenia,^[S15, S16]^ resulting in lower muscle strength and lower muscle mass. In our study, age, sex, hypertension, and diabetes were balanced across groups, avoiding biases that these factors may cause. Some studies have found that lifestyle is closely related to muscle condition; for example, smoking and drinking can accelerate muscle loss in patients,^[S17, S18]^ and prolonged sleep increases the risk of sarcopenia.^[S19]^ More frequent exercise could prevent frailty and may benefit sarcopenia prevention.^35^ Although no significant differences among patients were observed in terms of smoking status, drinking status, sleeping habits, or physical activity level in our study, most of the patients in Group A were vegetarians with little or no meat intake so vegetarianism may be a characteristic of sarcopenia. Studies have shown that low nutrient levels can lead to low haematopoietic function^36^; therefore, the low RBC count and slow recovery in Group A patients may be related to their low nutritional status.

The extent of sarcopenia is a strong prognostic factor for cancer, and a worse skeletal muscle condition is related to a worse prognosis.^10^, ^37^ Therefore, reducing muscle loss or increasing muscle mass through pre‐operative interventions can improve the prognosis of cancer patients. Some studies have found that reducing the consumption of skeletal muscle before surgery can be achieved in a short time before surgery by leucine/creatine supplementation and other methods. A leucine diet (27% of calories as total protein, 44% of calories as carbohydrate, and 30% of calories as fat, with fish oil, high protein, leucine, and specific oligosaccharides) consumed before surgery can effectively strengthen muscle synthesis in a few hours^26^ and maintain long‐lasting effects.^38^ These competitive pre‐operative nutritional choices are also suitable for cancer patients who have undergone limited operations. For patients who undergo elective surgery or receive adjuvant therapy, short‐term and medium‐term creatine therapy can effectively increase muscle strength,^39^ and 30–40 min of exercise three to four times a week or 2000 extra steps daily over 2–4 months or nutritional support can significantly decrease sarcopenia.^20^, ^24^, ^25^ Therefore, early detection and targeted interventions for sarcopenic patients in the HCC population before surgery are meaningful and necessary to improve post‐operative outcomes.

This is the first study thus far to investigate the effect of sarcopenia on short‐term post‐operative outcomes in HCC patients based on the EWGSOP,^16^ combining muscle mass and muscle strength. The number of HCC cases involved in this study is advantageous when solely comparing the number of HCCs (*Table* S1). Such a large sample size is still rare, whether retrospective or prospective. However, this study has a certain limitation. That is, it included liver cirrhosis patients and non‐cirrhosis patients. Although the groups were well balanced, it is still necessary to conduct further research on patients with or without cirrhosis separately in the future to guide targeted differential interventions.

## Conclusions

Sarcopenia is associated with poor short‐term outcomes after hepatectomy in patients with primary HCC and is an independent risk factor for major post‐operative complications. Assessments combining muscle mass and muscle strength at the first clinical visit can accurately identify sarcopenia patients. They may provide an effective time window for drug or nutritional intervention to improve short‐term post‐operative outcomes.

## Conflict of interest

The authors report no conflicts of interest related to this work.

## Funding

This study was supported by the National Natural Science Foundation of China (81772628, 81703310, 82072685) and the Research Foundation of National Health Commission of China–Major Medical and Health Technology Project for Zhejiang Province (WKJ‐ZJ‐1706).

## Supporting information


**Table S1.** Previous studies related to sarcopenia and liver cancer
**Table S2**. Haematological indicators before operation of the 3 groups
**Table S3**. Haematological indicators after operation of the 3 groups
**Table S4**. Logistic regression for predictive factors of complication

## References

[1] Sung H , Ferlay J , Siegel RL , Laversanne M , Soerjomataram I , Jemal A , Bray F . Global cancer statistics 2020: GLOBOCAN estimates of incidence and mortality worldwide for 36 cancers in 185 countries. CA Cancer J Clin 2021;71:209–249. 33538338 10.3322/caac.21660

[2] Knox JJ , Cleary SP , Dawson LA . Localized and systemic approaches to treating hepatocellular carcinoma. J Clin Oncol 2015;33:1835–1844. 25918289 10.1200/JCO.2014.60.1153

[3] Dimick JB , Wainess RM , Cowan JA , Upchurch GR Jr , Knol JA , Colletti LM . National trends in the use and outcomes of hepatic resection. J Am Coll Surg 2004;199:31–38. 15217626 10.1016/j.jamcollsurg.2004.03.005

[4] Jarnagin WR , Gonen M , Fong Y , DeMatteo RP , Ben‐Porat L , Little S , Corvera C , Weber S , Blumgart LH . Improvement in perioperative outcome after hepatic resection: analysis of 1,803 consecutive cases over the past decade. Ann Surg 2002;236:397–406, discussion 406‐407. 12368667 10.1097/01.SLA.0000029003.66466.B3 PMC1422593

[5] Stewart GD , O'Súilleabháin CB , Madhavan KK , Wigmore SJ , Parks RW , Garden OJ . The extent of resection influences outcome following hepatectomy for colorectal liver metastases. Eur J Surg Oncol 2004;30:370–376. 15063889 10.1016/j.ejso.2004.01.011

[6] Katz SC , Shia J , Liau KH , Gonen M , Ruo L , Jarnagin WR , Fong Y , D'Angelica MI , Blumgart LH , DeMatteo RP . Operative blood loss independently predicts recurrence and survival after resection of hepatocellular carcinoma. Ann Surg 2009;249:617–623. 19300227 10.1097/SLA.0b013e31819ed22f

[7] Ariizumi SI , Katagiri S , Kotera Y , Yamashita S , Omori A , Kato T , Egawa H , Takasaki K , Yamamoto M . Improved mortality, morbidity and long‐term outcome after anatomical hepatectomy with the Glissonean pedicle approach in patients with hepatocellular carcinoma: 30 years' experience at a single institute. Ann Surg 2020;23:S42–S43. 10.1097/SLA.0000000000004311 33273356

[8] Newman KL , Johnson KM , Cornia PB , Wu P , Itani K , Ioannou GN . Perioperative evaluation and management of patients with cirrhosis: Risk assessment, surgical outcomes, and future directions. Clin Gastroenterol Hepatol 2020;18:2398–2414.e3. 31376494 10.1016/j.cgh.2019.07.051 PMC6994232

[9] Cescon M , Vetrone G , Grazi GL , Ramacciato G , Ercolani G , Ravaioli M , del Gaudio M , Pinna AD . Trends in perioperative outcome after hepatic resection: analysis of 1500 consecutive unselected cases over 20 years. Ann Surg 2009;249:995–1002. 19474679 10.1097/SLA.0b013e3181a63c74

[10] Martin L , Birdsell L , Macdonald N , Reiman T , Clandinin MT , McCargar LJ , MacDonald N , Murphy R , Ghosh S , Sawyer MB , Baracos VE . Cancer cachexia in the age of obesity: Skeletal muscle depletion is a powerful prognostic factor, independent of body mass index. J Clin Oncol 2013;31:1539–1547. 23530101 10.1200/JCO.2012.45.2722

[11] Voron T , Tselikas L , Pietrasz D , Pigneur F , Laurent A , Compagnon P , Salloum C , Luciani A , Azoulay D . Sarcopenia impacts on short‐ and long‐term results of hepatectomy for hepatocellular carcinoma. Ann Surg 2015;261:1173–1183. 24950264 10.1097/SLA.0000000000000743

[12] Iritani S , Imai K , Takai K , Hanai T , Ideta T , Miyazaki T , Suetsugu A , Shiraki M , Shimizu M , Moriwaki H . Skeletal muscle depletion is an independent prognostic factor for hepatocellular carcinoma. J Gastroenterol 2015;50:323–332. 24817668 10.1007/s00535-014-0964-9

[13] Imamura H , Seyama Y , Kokudo N , Maema A , Sugawara Y , Sano K , Takayama T , Makuuchi M . One thousand fifty‐six hepatectomies without mortality in 8 years. Arch Surg 2003;138:1198–1206, discussion 1206. 14609867 10.1001/archsurg.138.11.1198

[14] Horowitz M , Neeman E , Sharon E , Ben‐Eliyahu S . Exploiting the critical perioperative period to improve long‐term cancer outcomes. Nat Rev Clin Oncol 2015;12:213–226. 25601442 10.1038/nrclinonc.2014.224 PMC5497123

[15] Schlagenhauff B , Ellwanger U , Breuninger H , Stroebel W , Rassner G , Garbe C . Prognostic impact of the type of anaesthesia used during the excision of primary cutaneous melanoma. Melanoma Res 2000;10:165–169. 10803717

[16] Cruz‐Jentoft AJ , Bahat G , Bauer J , Boirie Y , Bruyère O , Cederholm T , Cooper C , Landi F , Rolland Y , Sayer AA , Schneider SM , Sieber CC , Topinkova E , Vandewoude M , Visser M , Zamboni M , Writing Group for the European Working Group on Sarcopenia in Older People 2 (EWGSOP2), and the Extended Group for EWGSOP2 , Bautmans I , Baeyens JP , Cesari M , Cherubini A , Kanis J , Maggio M , Martin F , Michel JP , Pitkala K , Reginster JY , Rizzoli R , Sánchez‐Rodríguez D , Schols J . Sarcopenia: Revised European consensus on definition and diagnosis. Age Ageing 2019;48:16–31. 30312372 10.1093/ageing/afy169 PMC6322506

[17] Bone AE , Hepgul N , Kon S , Maddocks M . Sarcopenia and frailty in chronic respiratory disease. Chron Respir Dis 2017;14:85–99. 27923981 10.1177/1479972316679664 PMC5720213

[18] Ebadi M , Montano‐Loza AJ . Clinical relevance of skeletal muscle abnormalities in patients with cirrhosis. Dig Liver Dis 2019;51:1493–1499. 31221549 10.1016/j.dld.2019.05.034

[19] Nakamura R , Inage Y , Tobita R , Yoneyama S , Numata T , Ota K , Yanai H , Endo T , Inadome Y , Sakashita S , Satoh H , Yuzawa K , Terashima T . Sarcopenia in resected NSCLC: Effect on postoperative outcomes. J Thorac Oncol 2018;13:895–903. 29751134 10.1016/j.jtho.2018.04.035

[20] Berardi G , Antonelli G , Colasanti M , Meniconi R , Guglielmo N , Laurenzi A , Ferretti S , Levi Sandri GB , Spagnoli A , Moschetta G , Schininà V , Antonini M , Marignani M , Ettorre GM . Association of sarcopenia and body composition with short‐term outcomes after liver resection for malignant tumors. JAMA Surg 2020;155:e203336. 32965483 10.1001/jamasurg.2020.3336 PMC7512123

[21] Miyamoto Y , Baba Y , Sakamoto Y , Ohuchi M , Tokunaga R , Kurashige J , Hiyoshi Y , Iwagami S , Yoshida N , Yoshida M , Watanabe M , Baba H . Sarcopenia is a negative prognostic factor after curative resection of colorectal cancer. Ann Surg Oncol 2015;22:2663–2668. 25564158 10.1245/s10434-014-4281-6

[22] Otsuji H , Yokoyama Y , Ebata T , Igami T , Sugawara G , Mizuno T , Yamaguchi J , Nagino M . Surgery‐related muscle loss and its association with postoperative complications after major hepatectomy with extrahepatic bile duct resection. World J Surg 2017;41:498–507. 27718001 10.1007/s00268-016-3732-6

[23] van Wijk L , van Duinhoven S , Liem M , Bouman DE , Viddeleer AR , Klaase JM . Risk factors for surgery‐related muscle quantity and muscle quality loss and their impact on outcome. Eur J Med Res 2021;26:36. 33892809 10.1186/s40001-021-00507-9 PMC8063361

[24] Zenith L , Meena N , Ramadi A , Yavari M , Harvey A , Carbonneau M , Ma M , Abraldes JG , Paterson I , Haykowsky MJ , Tandon P . Eight weeks of exercise training increases aerobic capacity and muscle mass and reduces fatigue in patients with cirrhosis. Clin Gastroenterol Hepatol 2014;12:1920, e2–1926. 24768811 10.1016/j.cgh.2014.04.016

[25] Prado CM , Purcell SA , Laviano A . Nutrition interventions to treat low muscle mass in cancer. J Cachexia Sarcopenia Muscle 2020;11:366–380. 31916411 10.1002/jcsm.12525 PMC7113510

[26] Deutz NE , Safar A , Schutzler S , Memelink R , Ferrando A , Spencer H , van Helvoort A , Wolfe RR . Muscle protein synthesis in cancer patients can be stimulated with a specially formulated medical food. Clin Nutr 2011;30:759–768. 21683485 10.1016/j.clnu.2011.05.008 PMC3964623

[27] Kobayashi A , Kaido T , Hamaguchi Y , Okumura S , Shirai H , Yao S , Kamo N , Yagi S , Taura K , Okajima H , Uemoto S . Impact of sarcopenic obesity on outcomes in patients undergoing hepatectomy for hepatocellular carcinoma. Ann Surg 2019;269:924–931. 29064889 10.1097/SLA.0000000000002555

[28] Zhang G , Meng S , Li R , Ye J , Zhao L . Clinical significance of sarcopenia in the treatment of patients with primary hepatic malignancies, a systematic review and meta‐analysis. Oncotarget 2017;8:102474–102485. 29254263 10.18632/oncotarget.19687 PMC5731973

[29] Leal VO , Mafra D , Fouque D , Anjos LA . Use of handgrip strength in the assessment of the muscle function of chronic kidney disease patients on dialysis: A systematic review. Nephrol Dial Transplant 2011;26:1354–1360. 20709742 10.1093/ndt/gfq487

[30] Chen LK , Woo J , Assantachai P , Auyeung TW , Chou MY , Iijima K , Jang HC , Kang L , Kim M , Kim S , Kojima T , Kuzuya M , Lee JSW , Lee SY , Lee WJ , Lee Y , Liang CK , Lim JY , Lim WS , Peng LN , Sugimoto K , Tanaka T , Won CW , Yamada M , Zhang T , Akishita M , Arai H . Asian Working Group for Sarcopenia: 2019 consensus update on sarcopenia diagnosis and treatment. J Am Med Dir Assoc 2020;21:300–307.e2. 32033882 10.1016/j.jamda.2019.12.012

[31] Leong DP , Teo KK , Rangarajan S , Lopez‐Jaramillo P , Avezum A Jr , Orlandini A , Seron P , Ahmed SH , Rosengren A , Kelishadi R , Rahman O , Swaminathan S , Iqbal R , Gupta R , Lear SA , Oguz A , Yusoff K , Zatonska K , Chifamba J , Igumbor E , Mohan V , Anjana RM , Gu H , Li W , Yusuf S . Prognostic value of grip strength: Findings from the prospective urban rural epidemiology (PURE) study. Lancet 2015;386:266–273. 25982160 10.1016/S0140-6736(14)62000-6

[32] Schaap LA , van Schoor NM , Lips P , Visser M . Associations of sarcopenia definitions, and their components, with the incidence of recurrent falling and fractures: The longitudinal aging study Amsterdam. J Gerontol A Biol Sci Med Sci 2018;73:1199–1204. 29300839 10.1093/gerona/glx245

[33] Daly LE , Ní BÉB , Power DG , Cushen SJ , James K , Ryan AM . Loss of skeletal muscle during systemic chemotherapy is prognostic of poor survival in patients with foregut cancer. J Cachexia Sarcopenia Muscle 2018;9:315–325. 29318756 10.1002/jcsm.12267 PMC5879982

[34] Huang CY , Yang YC , Chen TC , Chen JR , Chen YJ , Wu MH , Jan YT , Chang CL , Lee J . Muscle loss during primary debulking surgery and chemotherapy predicts poor survival in advanced‐stage ovarian cancer. J Cachexia Sarcopenia Muscle 2020;11:534–546. 31999069 10.1002/jcsm.12524 PMC7113537

[35] Oliveira JS , Pinheiro MB , Fairhall N , Walsh S , Chesterfield Franks T , Kwok W , Bauman A , Sherrington C . Evidence on physical activity and the prevention of frailty and sarcopenia among older people: A systematic review to inform the World Health Organization physical activity guidelines. J Phys Act Health 2020;17:1247–1258. 32781432 10.1123/jpah.2020-0323

[36] Santos EW , Oliveira DC , Silva GB , Tsujita M , Beltran JO , Hastreiter A , Fock RA , Borelli P . Hematological alterations in protein malnutrition. Nutr Rev 2017;75:909–919. 29025154 10.1093/nutrit/nux041

[37] Hutchinson L . Risk factors: Cachexia‐‐skeletal muscle depletion is a prognostic factor. Nat Rev Clin Oncol 2013;10:250. 23568419 10.1038/nrclinonc.2013.57

[38] Leenders M , van Loon LJ . Leucine as a pharmaconutrient to prevent and treat sarcopenia and type 2 diabetes. Nutr Rev 2011;69:675–689. 22029833 10.1111/j.1753-4887.2011.00443.x

[39] Kley RA , Tarnopolsky MA , Vorgerd M . Creatine for treating muscle disorders. Cochrane Database Syst Rev 2013;2013:CD004760. 10.1002/14651858.CD004760.pub4 PMC6492334 23740606

[40] von Haehling S , Coats A , Anker SD . Ethical guidelines for publishing in the Journal of Cachexia, Sarcopenia and Muscle: Update 2021. J Cachexia Sarcopenia Muscle 2021;12:2259–2261. 34904399 10.1002/jcsm.12899 PMC8718061

