# Supplementary material for: Impact of sarcopenia on outcomes of patients undergoing liver resection for hepatocellular carcinoma
Source: J Cachexia Sarcopenia Muscle. 2022 Jul 19;13(5):2383–92. doi: 10.1002/jcsm.13040 (PMC9530540; doi:10.1002/jcsm.13040)
Supplement: Supplementary file 1 — Table S1. Previous studies related to sarcopenia and liver cancer Table S2. Haematological indicators before operation of the 3 groups Table S3. Haematological indicators after operation of the 3 groups Table S4. Logistic regression for predictive factors of complication [file JCSM-13-2383-s001.docx]

**Table S1.** Previous studies related to sarcopenia and liver cancer

| **Articles** | **Authors** | **Publication Date** | **DOI** | **Journal** | **Number of HCC** | **Study type** |
| --- | --- | --- | --- | --- | --- | --- |
| Sarcopenia Impacts on Short- and Long-term Results of Hepatectomy for Hepatocellular Carcinoma | Thibault Voron, et al. | 2015 Jun | 10.1097/SLA.0000000000000743 | Ann Surg | 109 | retrospective study |
| Impact of body composition on survival and morbidity after liver resection in hepatocellular carcinoma patients | Andreas Kroh, et al. | 2019 Feb | 10.1016/j.hbpd.2018.07.008 | Hepatobiliary Pancreat Dis Int | 70 | retrospective study |
| Impact of Sarcopenia as a Prognostic Factor on Reductive Hepatectomy for Advanced Hepatocellular Carcinoma | Satoshi Omiya, et al. | 2021 Nov | 10.21873/anticanres.15394 | Anticancer Res | 93 | retrospective study |
| Sarcopenia increases 1-year mortality after surgical resection of hepatocellular carcinoma | Mohamed A Salman, et al. | 2020 May | 10.1111/ans.15647 | ANZ J Surg | 52 | retrospective study |
| Sarcopenia predicts reduced survival in patients with hepatocellular carcinoma at first diagnosis | Paola Begini, et al. | 2017 Jan-Feb | 10.5604/16652681.1226821 | Ann Hepatol | 92 | retrospective study |
| Impact of body composition on survival and morbidity after liver resection in hepatocellular carcinoma patients. | Andreas Kroh, et al. | 2019 Feb | 10.1016/j.hbpd.2018.07.008 | Hepatobiliary Pancreat Dis Int | 70 | retrospective study |
| Impact of Sarcopenic Obesity on Outcomes in Patients Undergoing Hepatectomy for Hepatocellular Carcinoma | Kobayashi, Atsushi MD, et al. | 2019 May | 10.1097/SLA.0000000000002555 | Ann Surg | 465 | retrospective study |
| Sarcopenia as a predictor of prognosis in patients following hepatectomy for hepatocellular carcinoma | N Harimoto, et al. | 2013 Oct | 10.1002/bjs.9258 | Br J Surg | 186 | retrospective study |
| Sarcopenia negatively affects preoperative total functional liver volume in patients undergoing liver resection | Dello, et al. | 2013 Mar | 10.1111/j.1477-2574.2012.00517.x | HPB | 2 | retrospective study |
| Sarcopenia impairs survival in patients with potentially curable hepatocellular carcinoma | Levolger,et al. | 2015 Aug | 10.1002/jso.23976 | JOURNAL OF SURGICAL ONCOLOGY | 90 | retrospective study |
| Preoperative Sarcopenia Negatively Impacts Postoperative Outcomes Following Major Hepatectomy with Extrahepatic Bile Duct Resection | Hidehiko Otsuji, et al. | 2015 Feb | 10.1007/s00268-015-2988-6 | World Journal of Surgery | 3 | retrospective study |
| Association of Sarcopenia and Body Composition With Short-term Outcomes After Liver Resection for Malignant Tumors | Giammauro Berardi, et al. | 2020 Nov | 10.1001/jamasurg.2020.3336 | JAMA Surg | 101 | perspective study |

**Table S2**. Hematological indicators before operation of the 3 groups

|  | **Total (n=155)** | **Group A (n=22)** | **Group B (n=67)** | **Group C (n=66)** | **P value** |
| --- | --- | --- | --- | --- | --- |
| AST, U/L | 26.00 (18.00-44.50) | 23.00 (15.00-47.75) | 25.00 (18.00-49.50) | 30.50 (19.00-43.00) | 0.471 |
| ALT, U/L | 30.00 (23.75-43.40) | 29.00 (22.00-49.25) | 28.50 (23.75-42.25) | 31.00 (24.00-39.50) | 0.849 |
| GGT , U/L | 50.00 (28.00-99.80) | 99.80 (47.50-160.50) | 56.00 (31.00-91.80) | 41.50 (26.75-91.80) | 0.165 |
| Cholesterol, mmol/L | 4.68 ± 0.80 | 4.32 ± 0.28 | 4.77 ± 0.11 | 4.71 ± 0.12 | 0.185 |
| TBIL, Umol/L | 10.00 (8.00-14.00) | 10.00 (9.00-14.50) | 10.50 (8.00-16.25) | 10.00 (8.00-14.00) | 0.475 |
| Cr, Umol/L | 72.00 (64.75-83.00) | 70.00 (64.25-84.00) | 73.00 (66.00-82.00) | 72.50 (61.00-85.25) | 0.924 |
| ALB, g/L | 40.25 (37.65-43.40) | 37.85 (34.55-40.93) | 41.30 (38.10-43.30) | 40.35 (38.13-43.95) | 0.025 |
| TG, mmol/L | 1.24 (0.90-1.69) | 1.03 (0.73-1.72) | 1.16 (0.91-1.62) | 1.43 (1.06-1.94) | 0.068 |
| HDL, mmol/L | 1.01 (0.86-1.21) | 1.04 (0.90-1.31) | 1.02 (0.86-1.22) | 0.98 (0.81-1.07) | 0.122 |
| LDL, mmol/L | 2.60 (2.06-3.07) | 2.06 (1.71-2.94) | 2.60 (2.06-3.20) | 2.62 (2.17-3.04) | 0.182 |
| LDH, U/L | 202.01 (182.00-209.00) | 202.01 (177.25-212.25) | 202.01 (175.00-207.00) | 202.01 (187.25-213.25) | 0.332 |
| INR | 1.02 (0.98-1.07) | 1.05 (0.99-1.13) | 1.02 (0.97-1.08) | 1.02 (0.98-1.06) | 0.469 |
| PT, s | 13.30 (13.00-13.90) | 13.45 (13.08-14.30) | 13.30 (12.80-13.90) | 13.40 (12.98-13.8) | 0.417 |
| AFP, ng/mL |  |  |  |  | 0.567 |
| <=400 | 131 | 17 | 58 | 56 |  |
| >400 | 24 | 5 | 9 | 10 |  |
| CEA, Ug/L | 2.10 (1.40-2.80) | 2.15 (1.55-3.08) | 2.10 (1.40-2.80) | 2.15 (1.40-3.20) | 0.865 |
| CA199, U/mL | 15.60 (7.20-22.90) | 21.07 (10.53-31.48) | 12.00 (6.60-21.70) | 18.90 (7.18-27.03) | 0.020 |
| WBC, ×10^9^/L | 5.91 (4.77-6.93) | 5.10 (3.92-8.10) | 5.68 (4.79-6.73) | 6.22 (4.94-7.37) | 0.283 |
| RBC, ×10^12^/L | 4.60 ± 0.43 | 4.45 ± 0.11 | 4.57 ± 0.07 | 4.68 ± 0.06 | 0.173 |
| NEUT, ×10^9^/L | 3.66 (2.75-4.56) | 2.88 (1.94-4.06) | 3.66 (2.86-4.47) | 3.95 (2.77-5.01) | 0.046 |
| Monocyte, ×10^9^/L | 0.43 (0.35-0.59) | 0.42 (0.31-0.61) | 0.42 (0.35-0.62) | 0.47 (0.37-0.58) | 0.861 |
| HGB, g/L | 147.00 (134.00-154.00) | 134.00 (126.25-150.00) | 148.00 (134.00-155.00) | 147.50 (134.75-156.25) | 0.041 |
| PLT, ×10^9^/L | 178.00(139.00-229.00) | 173.00 (126.75-200.00) | 175.00 (137.00-232.0) | 181.00 (148.00-230.00) | 0.251 |
| Fib, g/L | 2.74 (2.38-3.18) | 2.83 (2.29-4.16) | 2.75 (2.43-3.23) | 2.72 (2.35-3.08) | 0.496 |

ALT, alanine aminotransferase; AST, aspartate aminotransferase; GGT, γ-glutamyltransferase; TBIL, total bilirubin; Cr, creatinine; ALB, albumin; TG, triglyceride; HDL, high density lipoprotein; LDL, low density lipoprotein; LDH, Lactate dehydrogenase; INR, international normalized ratio; PT, Prothrombin time; AFP, alpha-fetoprotein; CEA, carcinoembryonic antigen; CA199, Saccharide antigen 199; WBC, white blood cell; RBC, red blood cell; NEUT, neutrophil; HGB, hemoglobin; PLT, blood platelet; Fib, Fibrinogen.

**Table S3**. Hematological indicators after operation of the 3 groups

|  | **Total (n=155)** | **Group A (n=22)** | **Group B (n=67)** | **Group C (n=66)** | **P value** |
| --- | --- | --- | --- | --- | --- |
| **The first day after operation** | | | | | |
| ALT, U/L | 299.00 (156.00-580.00) | 427.00 (228.50-553.00) | 321.00 (201.00-501.50) | 362.00 (127.50-614.25) | 0.434 |
| AST, U/L | 344.50 (233.75-569.50) | 320.50 (239.00-596.00) | 216.00 (220.00-503.00) | 221.25 (122.25-680.75) | 0.259 |
| TBIL, Umol/L | 20.00 (16.00-29.25) | 24.00 (15.50-31.00) | 20.50 (17.00-31.25) | 19.00 (15.00-24.00) | 0.489 |
| ALB, g/L | 33.81 ± 0.46 | 31.44 ± 1.17 | 33.55 ± 0.67 | 35.35 ± 0.66 | <0.001 |
| Cr, Umol/L | 66.00 (58.00-82.00) | 67.00 (59.00-86.50) | 67.00 (58.00-88.00) | 65.00 (55.00-75.00) | 0.608 |
| INR | 1.17 (1.10-1.24) | 1.22 (1.15-1.31) | 1.19 (1.07-1.22) | 1.15 (1.09-1.20) | 0.185 |
| PT, s | 14.90 (14.20-15.80) | 15.80 (14.60-16.80) | 14.90 (14.00-15.55) | 14.70 (14.18-15.18) | 0.215 |
| WBC, ×10^9^/L | 11.37 (9.16-13.83) | 11.54 (9.78-13.59) | 11.27 (8.47-14.28) | 11.59 (9.33-14.87) | 0.633 |
| PLT, ×10^9^/L | 141.64 ± 6.76 | 116.18 ± 10.03 | 146.73 ± 10.08 | 148.81 ± 12.63 | 0.095 |
| RBC, ×10^12^/L | 3.93 ± 0.73 | 3.54 ± 0.19 | 3.92 ± 0.10 | 4.15 ± 0.11 | 0.003 |
| **The seventh day after operation** | | | | | |
| ALT, U/L | 73.00 (41.00-120.00) | 77.00 (45.50-136.00) | 59.50 (38.25-103.25) | 107.00 (40.25-139.00) | 0.530 |
| AST, U/L | 30.00 (24.00-35.75) | 28.00 (23.50-52.00) | 37.00 (23.00-33.00) | 31.00 (26.50-42.50) | 0.118 |
| TBIL, Umol/L | 15.00 (11.00-21.00) | 20.00 (13.50-29.00) | 14.00 (11.00-19.75) | 15.00 (9.50-21.00) | 0.015 |
| ALB, g/L | 34.77 ± 0.63 | 33.89 ± 1.06 | 34.40 ± 0.85 | 35.66 ± 1.26 | 0.649 |
| Cr, Umol/L | 63.00 (55.00-76.00) | 58.00 (50.50-67.00) | 64.00 (57.00-78.00) | 62.00 (49.50-73.00) | 0.534 |
| INR | 1.14 (1.08-1.23) | 1.15 (1.07-1.28) | 1.16 (1.09-1.25) | 1.13 (1.07-1.19) | 0.149 |
| PT, s | 14.60 (14.10-15.40) | 14.40 (14.10-15.70) | 14.70 (14.13-15.90) | 14.60 (13.98-14.90) | 0.109 |
| WBC, ×10^9^/L | 6.93 (5.25-8.80) | 6.51 (4.88-8.71) | 7.34 (5.13-9.25) | 6.80 (5.38-8.14) | 0.922 |
| PLT, ×10^9^/L | 176.82 ± 8.64 | 133.82 ± 14.88 | 180.53 ± 12.27 | 195.03 ± 15.77 | 0.037 |
| RBC, ×10^12^/L | 3.58 ± 0.63 | 3.34 ± 0.13 | 3.54 ± 0.09 | 3.74 ± 0.11 | 0.003 |

**Table S4**. Logistic regression for predictive factors of complication

|  | **Univariate analysis** | | **Multivariate analysis** | |
| --- | --- | --- | --- | --- |
|  | **HR (95% CI)** | **P value** | **HR (95% CI)** | **P value** |
| Age (years) | 1.014(0.981-1.047） | 0.409 |  |  |
| Sex (male vs. female) | 1.355 (0.462-3.972） | 0.581 |  |  |
| BMI (Kg/m^2^) | 0.933（0.825-1.055） | 0.271 |  |  |
| Abdominal circumference (cm) | 0.992（0.947-1.039） | 0.729 |  |  |
| Visceral adipose tissue (cm^2^) | 1.000（1.000-1.000） | 0.773 |  |  |
| Subcutaneous adipose tissue (cm^2^) | 1.000（1.000-1.000） | 0.495 |  |  |
| Skeletal Muscle Index (cm/m^2^) | 0.982（0.948-1.017） | 0.305 |  |  |
| Grip strength test (Kg) | 0.964（0.925-1.004） | 0.077 |  |  |
| Chair stand test (s) | 1.050（0.958-1.152） | 0.299 |  |  |
| Gait speed (m/s) | 0.911（0.148-5.625） | 0.920 |  |  |
| Smoke (previous or current vs. never) | 1.358（0.657-2.803） | 0.409 |  |  |
| Drink (previous or current vs. never) | 1.186（0.596-2.361） | 0.627 |  |  |
| Physical activity (yes vs. no) | 0.829（0.400-1.718） | 0.614 |  |  |
| Dietary structure |  |  |  |  |
| Vegetarian | 1.546（0.628-3.808） | 0.343 |  |  |
| Semi-vegetarian | 1.188（0.542-2.605） | 0.667 |  |  |
| Meat eater |  | 1.000 |  |  |
| Sleep time (>=8h vs. <8h) | 0.935（0.415-2.109） | 0.871 |  |  |
| SARCF (>=4 vs. <4) | 1.182（0.735-1.900） | 0.490 |  |  |
| ASA grade |  |  |  |  |
| I |  | 1.000 |  |  |
| II | 0.514（0.112-2.359） | 0.392 |  |  |
| III | 1.593（0.438-5.794） | 0.480 |  |  |
| Cirrhosis (yes vs. no) | 1.358（0.657-2.803） | 0.409 |  |  |
| Ascites (yes vs. no) | 1.333（0.344-5.164） | 0.677 |  |  |
| Hypertension (yes vs. no) | 1.123（0.543-2.325） | 0.754 |  |  |
| Diabetes (yes vs. no) | 1.129（0.499-2.557） | 0.771 |  |  |
| Age-adjusted Charlson comorbidity score | 1.232（0.990-1.533） | 0.062 |  |  |
| Child-Pugh stage (B vs. A) | 1.092（0.204-5.842） | 0.918 |  |  |
| MELD score | 1.138（0.975-1.328） | 0.100 |  |  |
| BCLC stage |  |  |  |  |
| 0 |  | 1.000 |  |  |
| A | 2.032（0.732-5.642） | 0.174 |  |  |
| B | 0.444（0.060-3.285） | 0.427 |  |  |
| C | 1.333（0.449-3.959） | 0.604 |  |  |
| TNM stage (III-IV vs. I-II) | 1.718（0.516-5.719） | 0.378 |  |  |
| Microvascular invasion (yes vs. no) | 1.226（0.590-2.547） | 0.585 |  |  |
| Satellite stove (yes vs. no) | 1.610（0.538-4.813） | 0.394 |  |  |
| Lesion size | 1.087（0.974-1.212） | 0.136 |  |  |
| Lesions (multiple vs. solitary) | 1.290（0.447-3.723） | 0.637 |  |  |
| Differentiation of HCC | 1.310（0.747-2.297） | 0.346 |  |  |
| Poor | 1.667（0.506-5.494） | 0.401 |  |  |
| Moderate | 1.227（0.406-3.712） | 0.717 |  |  |
| Well |  | 1.000 |  |  |
| Reduced Muscle Mass and Strength | 12.000（3.886-37.061） | **<0.001** | 10.735(2.547-45.244) | **0.001** |
|  |  |  |  |  |
| Reduced Muscle Mass or Strength | 1.781（0.784-4.047） | 0.168 | 1.881(0.803-4.407) | 0.146 |
| Normal Muscle Mass and Strength |  | 1.000 |  | 1.000 |
| Operation mode (open vs. Laparoscopic) | 2.253（1.111-4.568） | **0.024** | 4.528(1.425-14.387) | **0.010** |
| Type of hepatectomy (Major vs. minor) | 1.198（0.603-2.380） | 0.606 |  |  |
| Operative time (min) | 1.003（0.998-1.009） | 0.236 |  |  |
| Blood loss (ml) (>400 vs. <= 400) | 1.003（0.997-1.009） | 0.296 |  |  |
| Hematological indicators of the first admission | | | | |
| AST (U/L) | 1.003（0.995-1.011） | 0.483 |  |  |
| ALT (U/L) | 1.005（0.996-1.013） | 0.278 |  |  |
| GGT level (U/L) | 1.001（0.999-1.003） | 0.271 |  |  |
| Cholesterol (mmol/L) | 0.761（0.531-1.089） | 0.135 |  |  |
| TBIL (Umol/L) | 1.038（0.987-1.092） | 0.147 |  |  |
| Cr (Umol/L) | 0.994（0.974-1.014） | 0.562 |  |  |
| ALB (g/L) | 0.958（0.900-1.021） | 0.185 |  |  |
| TG (mmol/L) | 0.683（0.432-1.082） | 0.104 |  |  |
| HDL (mmol/L) | 0.691（0.184-2.594） | 0.584 |  |  |
| LDL (mmol/L) | 0.828（0.531-1.291） | 0.405 |  |  |
| LDH (U/L) | 0.995（0.985-1.005） | 0.334 |  |  |
| INR | 16.293(0.251-1058.606） | 0.190 |  |  |
| PT (s) | 1.349（0.898-2.026） | 0.149 |  |  |
| AFP (ng/Ml) (>=400 vs. <400) | 1.026（0.411-2.557） | 0.957 |  |  |
| CEA (Ug/L) | 1.033（0.823-1.296） | 0.780 |  |  |
| CA199 (U/mL) | 1.004（0.989-1.019） | 0.611 |  |  |
| WBC level (10^9^/L) | 1.081（0.891-1.313） | 0.429 |  |  |
| RBC level (10^12^/L) | 0.853（0.450-1.619） | 0.628 |  |  |
| NEUT level (10^9^/L) | 0.101（0.880-1.378） | 0.399 |  |  |
| Monocyte level (10^9^/L) | 3.453（0.737-16.175） | 0.116 |  |  |
| HGB level (g/L) | 1.000（0.981-1.020） | 0.989 |  |  |
| PLT count (10^9^/L) | 0.998（0.993-1.003） | 0.387 |  |  |
| Fib level (g/L) | 1.549（1.029-2.333） | **0.036** | 1.278（0.808-2.022） | 0.294 |

**Supplementary References**

S1. Liao X, Zhang D. The 8th Edition American Joint Committee on Cancer Staging for Hepato-pancreato-biliary Cancer: A Review and Update. *Arch Pathol Lab Med* 2021;**145**:543-553.

S2. Koppie TM, Serio AM, Vickers AJ, Vora K, Dalbagni G, Donat SM, et al. Age-adjusted Charlson comorbidity score is associated with treatment decisions and clinical outcomes for patients undergoing radical cystectomy for bladder cancer. *Cancer* 2008;**112**:2384-2392.

S3. Kamath PS, Wiesner RH, Malinchoc M, Kremers W, Therneau TM, Kosberg CL, et al. A model to predict survival in patients with end-stage liver disease. *Hepatology* 2001;**33**:464-470.

S4. Reig M, Forner A, Rimola J, Ferrer-Fàbrega J, Burrel M, Garcia-Criado Á, et al. BCLC strategy for prognosis prediction and treatment recommendation: The 2022 update. *J Hepatol* 2021.

S5. Malmstrom TK, Miller DK, Simonsick EM, Ferrucci L, Morley JE. SARC-F: a symptom score to predict persons with sarcopenia at risk for poor functional outcomes. *J Cachexia Sarcopenia Muscle* 2016;**7**:28-36.

S6. Cesari M, Pahor M, Bartali B, Cherubini A, Penninx BW, Williams GR, et al. Antioxidants and physical performance in elderly persons: the Invecchiare in Chianti (InCHIANTI) study. *Am J Clin Nutr* 2004;**79**:289-294.

S7. Baines S, Powers J, Brown WJ. How does the health and well-being of young Australian vegetarian and semi-vegetarian women compare with non-vegetarians. *Public Health Nutr* 2007;**10**:436-442.

S8. Dindo D, Demartines N, Clavien PA. Classification of surgical complications: a new proposal with evaluation in a cohort of 6336 patients and results of a survey. *Ann Surg* 2004;**240**:205-213.

S9. Rahbari NN, Garden OJ, Padbury R, Brooke-Smith M, Crawford M, Adam R, et al. Posthepatectomy liver failure: a definition and grading by the International Study Group of Liver Surgery (ISGLS). *Surgery* 2011;**149**:713-724.

S10. Koch M, Garden OJ, Padbury R, Rahbari NN, Adam R, Capussotti L, et al. Bile leakage after hepatobiliary and pancreatic surgery: a definition and grading of severity by the International Study Group of Liver Surgery. *Surgery* 2011;**149**:680-688.

S11. Rahbari NN, Garden OJ, Padbury R, Maddern G, Koch M, Hugh TJ, et al. Post-hepatectomy haemorrhage: a definition and grading by the International Study Group of Liver Surgery (ISGLS). *HPB (Oxford)* 2011;**13**:528-535.

S12. Wakabayashi G, Cherqui D, Geller DA, Buell JF, Kaneko H, Han HS, et al. Recommendations for laparoscopic liver resection: a report from the second international consensus conference held in Morioka. *Ann Surg* 2015;**261**:619-629.

S13. Petermann-Rocha F, Balntzi V, Gray SR, Lara J, Ho FK, Pell JP, et al. Global prevalence of sarcopenia and severe sarcopenia: a systematic review and meta-analysis. *J Cachexia Sarcopenia Muscle* 2021.

S14. Lau EM, Lynn HS, Woo JW, Kwok TC, Melton LJ 3rd. Prevalence of and risk factors for sarcopenia in elderly Chinese men and women. *J Gerontol A Biol Sci Med Sci* 2005;**60**:213-216.

S15. Bai T, Fang F, Li F, Ren Y, Hu J, Cao J. Sarcopenia is associated with hypertension in older adults: a systematic review and meta-analysis. *BMC Geriatr* 2020;**20**:279.

S16. Veronese N, Pizzol D, Demurtas J, Soysal P, Smith L, Sieber C, et al. Association between sarcopenia and diabetes: a systematic review and meta-analysis of observational studies. *Eur Geriatr Med* 2019;**10**:685-696.

S17. Rom O, Kaisari S, Aizenbud D, Reznick AZ. Sarcopenia and smoking: a possible cellular model of cigarette smoke effects on muscle protein breakdown. *Ann N Y Acad Sci* 2012;**1259**:47-53.

S18. Han P, Kang L, Guo Q, Wang J, Zhang W, Shen S, et al. Prevalence and Factors Associated With Sarcopenia in Suburb-dwelling Older Chinese Using the Asian Working Group for Sarcopenia Definition. *J Gerontol A Biol Sci Med Sci* 2016;**71**:529-535.

S19. Nakakubo S, Doi T, Tsutsumimoto K, Kurita S, Ishii H, Shimada H. Sleep duration and progression to sarcopenia in Japanese community-dwelling older adults: a 4 year longitudinal study. *J Cachexia Sarcopenia Muscle* 2021;**12**:1034-1041.
